# Reparative Effects of Astaxanthin-Hyaluronan Nanoaggregates against Retrorsine-CCl_4_-Induced Liver Fibrosis and Necrosis

**DOI:** 10.3390/molecules23040726

**Published:** 2018-03-22

**Authors:** Yi Jhen Wu, Yu Chiuan Wu, I-Fen Chen, Yi-Lung Wu, Chin Wen Chuang, Han Hsiang Huang, Shyh Ming Kuo

**Affiliations:** 1 Department of Biomedical Engineering, I-Shou University, Kaohsiung City 82445, Taiwan; a9750069@gmail.com (Y.J.W.); ifen@isu.edu.tw (I.-F.C.); 2 Hualien Armed Forces General Hospital, Hualien County 97054, Taiwan; ranger.wu1113@gmail.com; 3 Department of Culinary Arts, National Kaohsiung University of Hospitality and Tourism, Kaohsiung City 81271, Taiwan; 4 Research & Development Department, Biored Technologies Inc., Tainan 70401, Taiwan; bioweiwang@yahoo.com.tw; 5 Department of Electrical Engineering, I-Shou University, Kaohsiung City 82445, Taiwan; cwchuang@isu.edu.tw; 6 Department of Veterinary Medicine, National Chiayi University, Chiayi 60054, Taiwan

**Keywords:** astaxanthin, hyaluronan nanoparticle, astaxanthin-hyaluronan nanoparticles-aggregate, liver fibrosis and necrosis

## Abstract

Astaxanthin (Asta), a xanthophyll carotenoid, has been reported to be a strong antioxidative agent and has anti-inflammatory, antitumor and free radical-scavenging activities. However, inadequate stability and water solubility results in its low bioavailability. This study incorporated Asta into hydrophilic hyaluronan nanoparticles (HAn) to produce Asta-HAn aggregates (AHAna) using an electrostatic field system and investigated the restorative effects of AHAna on retrorsine-CCl_4_-induced liver fibrosis in rats in vivo. Transmission electron microscopy (TEM) revealed that the prepared HAn were approximately 15 ± 2.1 nm in diameter and after the incorporation of Asta into HAn, the size increased to 210–500 nm. The incorporation efficiency of Asta was approximately 93% and approximately 54% of Asta was released after incubation for 18 h. Significant reductions in alanine aminotransferase and aspartate aminotransferase levels were observed after the rats were intraperitoneally injected with AHAna. Histopathological findings revealed the greatest reduction in hepatic fibrosis and hepatocyte necrosis in the rats after 2 weeks of intraperitoneal injection with AHAna, which is consistent with the data acquired from serum biochemical analysis. The restorative effects on liver damage displayed by AHAna in vivo demonstrated that Asta aggregated through HAn incorporation exerts therapeutic effects on liver fibrosis and necrosis.

## 1. Introduction

Astaxanthin (Asta) is a xanthophyll carotenoid found in various marine animals and algae including Haematococcus pluvialis. Asta is a powerful antioxidant agent and possesses anti-inflammatory, antitumor and free radical–scavenging activities [1]. Recently, Asta has been increasingly used as a medicinal ingredient to prevent and treat diseases such as cancer, age-related macular degeneration, inflammation and cardiovascular oxidative stress [2]. Asta has been reported to prevent diet-induced obesity and hepatic steatosis in mice and to ameliorate insulin resistance by decreasing oxidative stress. Furthermore, it possesses antioxidative activity that is 10 times greater than that of β-carotene [3]. However, Asta is unstable and decomposes drastically when exposed to heat, light, or oxygen and it exhibits unsatisfactory water solubility and dispersibility in aqueous solution (i.e., it yields low bioavailability). These features have prevented Asta from being used in pharmaceutical or biomedical applications. Numerous strategies have been employed to enhance Asta’s solubility and stability, such as encapsulating it into a hydrophilic polymer matrix and incorporating it into liposomes through emulsion or suspension [4,5]. 

The liver is one of the most multifunctional and important organs and the largest internal organ in the body. The liver has capacity to regenerate by the replication of mature liver cells. This regenerative response is clinically essential as the liver injured. However, when the liver suffers huge and/or long-term injury, such as an excess of uptake and constant exposure to hepatotoxic substances or drugs, the liver may progress into a stage of functional deficiency to overwhelm or even inhibit the intrinsic regeneration or liver tissue repair, resulting in severe consequences such as delayed repair in liver, liver fibrosis, liver failure or death. Hepatic tissues under these severe situations may be degenerative, necrotic and fibrotic. Hepatocytes consist of the majority of liver mass and execute the most functions of liver. Hepatocytes are mitosis inactive at normal status whereas they proliferate physiologically when damage occurs in the liver. Non-alcoholic fatty liver disease (NAFLD) is recognized as a major chronic liver disease in Asia [6] as liver fibrosis is a common cause leading to cirrhosis, which is the final fate of injury for the liver. Investigation has shown that patients with the active form of NAFLD, or called steatohepatitis (NASH), are probably coupled with fibrosis progression and eventually may result in cirrhosis [7]. CCl_4_ and retrorsine have been used to induce liver fibrosis/necrosis model in mice or rats [8,9,10]. Antioxidants, such as silymarin and vitamin E, have been selected as remedies for liver fibrosis and steatosis. The natural carotenoid compound astaxanthin (Asta) has also been found to potentially become a protector against liver pathologies [5,11].

Nanomedicine is a division of nanotechnology in which nanoparticles and nanoaggregates are applied to medicine. Numerous achievements in nanoparticle technology have enabled nanoparticle-based drug delivery systems to target tissues, pharmacokinetic profiles and administration routes. Nanoparticles used for drug delivery are submicron-sized (<1 μm) colloidal particles prepared using biocompatible and biodegradable materials. Hyaluronan (HA) is a hydrophilic, biodegradable, nontoxic and nonimmunogenic glycosaminoglycan. HA can be employed in various drug delivery methods, such as encapsulation and in various types of nanoparticles where it acts as a ligand for preparing platforms to actively target drugs, genes and diagnostic agents. In addition, lipid-based liposomes, micelles and emulsions, as well as other biopolymeric nanoparticles such as chitosan and gelatin, which possess advantageous biocompatible and biodegradable features, are specifically selected as carriers in the drug delivery system. These biodegradable nanoparticles provide favorable drug and vaccine encapsulation and convenient release profiles for numerous drugs, vaccines and biomolecules to enhance nanoparticle permeability, cellular uptake, bioavailability and retention. In our previous studies, we successfully fabricated HA nanoparticles (HAn) using an electrostatic field system (EFS) in an aqueous-phase environment to encapsulate drugs and demonstrated that HAn-derived drug aggregates have antitumor effects. Evidence has further shown that aggregation/encapsulation of HA nanoparticles enhances antitumor effects of potential compounds and drugs [12,13,14,15]. HA is an anionic, nonsulfated, water-soluble polysaccharide and it is biologically essential to cellular functions. As a drug delivery carrier, HA has numerous functional groups obtainable for conjugation. In this study, Asta was aggregated with HAn by using the EFS method. The effects of pure Asta, HAn and Asta–HAn aggregates (AHAna) on liver fibrosis and necrosis were investigated in vivo in a rat model.

## 2. Materials and Methods

### 2.1. Materials

Astaxanthin was produced and provided by Bioptik Technology Inc. (Miaoli, Taiwan). Asta extraction was performed according to the method described in the US patent US8030523B2, filed by Bioptik Technology Inc. Briefly, Pomacae canaliculata eggs were mixed proportionally with deionized water, homogenized (Polytron PT-2100, Bestway, Taipei, Taiwan) and the egg shells were removed to obtain a glycoprotein carotenoid solution. Then the protein, sugar and lipid in the glycoprotein carotenoid solution were removed sequentially to obtain the carotenoid solution. Finally, the pure Asta was extracted using 95% ethanol and the purity of the Asta was measured using the method described by Skrede et al [16]. Hyaluronan, dimethyl sulfoxide (DMSO) and FeCl_3_ were obtained from Sigma (St. Louis, MO, USA). All chemicals used in this study were of reagent grade. Retrorsine and CCl_4_ were purchased from Sigma.

### 2.2. Production and Characterization of Asta-HA Nano-Aggregates (AHAna)

**Figure 1:**
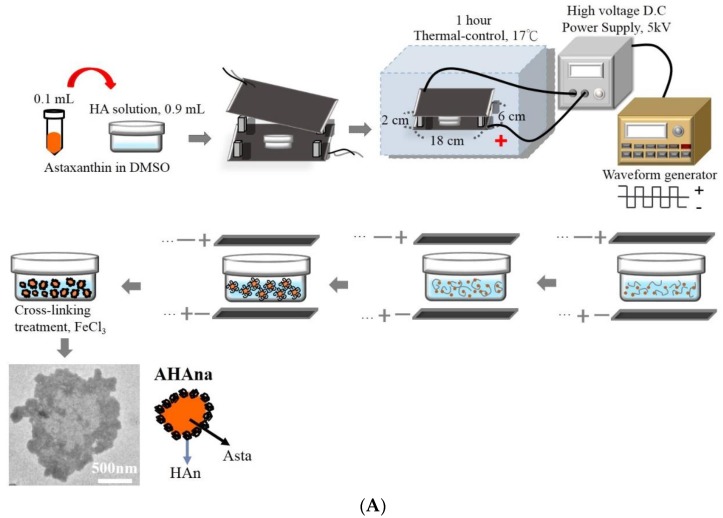

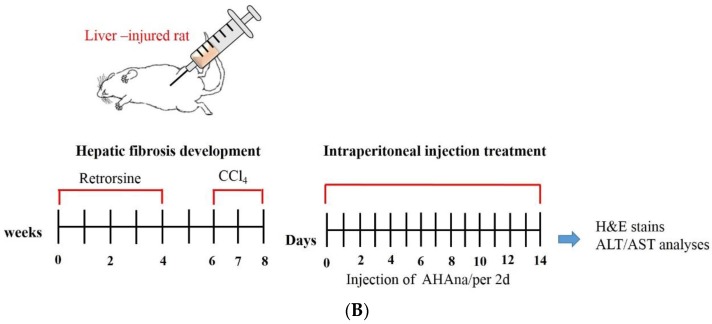
The schematic diagrams representing the preparation processes of AHAna aggregates (**A**) and the procedures and timescale for developing the rat liver injured mode (**B**).

HAn and AHAna were fabricated in an electrostatic field system (EFS) according to our previous reports [14]. Briefly, a stock solution of 0.2 mg/mL was prepared by dissolving Asta in dimethyl sulfoxide (DMSO). 0.9 mL HA solution (0.2 mg/mL) was carefully mixed with 0.1 mL Asta solution. The mixture was transferred onto a petri dish and placed at the center between the two plate electrodes. The preparation parameters of AHAna in the EFS were as follows: temperature: 17 °C, applied electric field strength: 5.0 kV/cm, reaction time: 1 h and crosslinking reagents: 0.001 N FeCl_3_. A schematic of the preparation procedures and the transmission electron microscopy (TEM) images of HAn and AHAna are shown in Figure 1. 

### 2.3. Incorporation Efficiency

The incorporation efficiency (IE) of Asta in the AHAna was determined as follows: 1 mL of solution containing the prepared AHAna was added to a centrifuge tube and was subsequently placed on a 40-rpm shaker at 37 °C. The sample was centrifuged at 14,000 rpm for 60 min before the amount of nonencapsulated Asta in the supernatant was used to determine the amount of Asta through high-performance liquid chromatography (HPLC, Agilent 1100 series; Agilent, Santa Clara, CA, USA). The IE of Asta in HAn aggregates was calculated using the following formula:

IE (%) = [(total amount of Asta − amount of nonencapsulated Asta)/total amount of Asta)] × 100%

### 2.4. In Vitro Release Study

The in vitro release of Asta from AHAna was investigated by placing 1 mL of AHAna into a 1.5-mL microcentrifuge tube. The tube was subsequently placed on a 40-rpm shaker at 37 °C. At defined time points, the sample was centrifuged at 14,000 rpm for 60 min. Subsequently, the amount of nonencapsulated Asta in the supernatant was determined through HPLC (Agilent). The experiments were performed in triplicate.

In vitro release (%) = [(total amount of Asta − residue of Asta)/Total amount of Asta] × 100%

### 2.5. In Vitro Cell Viability Study of Asta on L929 Fibroblasts

The effects of Asta on L929 fibroblast viability were assessed through MTT assay. The cells (1 × 104 cells/well) were seeded in a 96-well plate for 24 h and were subsequently treated with various concentrations of Asta. After 24 h and 48 h incubation, 20 μL of MTT solution (5 mg/mL) was added to the cells, which were incubated for an additional 4 h. The formazan precipitate was dissolved in DMSO and absorbance was obtained on a multiplate reader at 570 nm (Thermo Scientific, Waltham, MA, USA). Cells grown in a cell culture flask filled with DMSO in medium served as the control group.

### 2.6. Animals and Experimental Design

Animal studies were approved by the Institutional Animal Care and Use Committee of I-Shou University, Taiwan (AUP-105-50-01). Retrorsine working solution (10 mg/mL) was prepared in distilled water. The solution was titrated to pH 2.5 with 1 N HCl to completely dissolve retrorsine. Subsequently, the solution was neutralized using 1 N NaOH to yield a final concentration of 6 mg/mL. CCl_4_ was prepared by diluting the solution at a 1:9 (*v*/*v*) ratio with corn oil to yield a final concentration of 159.4 mg/mL. A total of 12 six-week-old male Sprague–Dawley rats were used in the fibrotic and necrotic liver injury model. The rats were randomized into the experimental groups (treated with retrorsine and CCl_4_) with necrotic or fibrotic liver injury (*n* = 9) and the control group (without treatment; *n* = 3). The rats in the experimental groups received two intraperitoneal injections (IP) of retrorsine (30 mg/kg) 2 weeks apart. Two weeks after the second retrorsine injection, the rats were administered two doses of CCl_4_ (0.4 g/kg, IP) 1 week apart. The rats were divided into three groups. Group B was the negative control group, in which the rats with liver injury received no treatment. Group C included the rats with liver injury that were treated with injections of pure HAn. Group D included the rats with liver injury that were treated with AHAna. The rats with liver injury that received pure Asta were designated as Group E.

In vivo experiments were conducted for 10 weeks. The first 8-week period was designed to allow the rats to develop liver fibrotic and necrotic injury and was followed by 2-weeks treatment with intraperitoneal injections of HAn, AHAna, or Asta into the injured livers (700 μL/2 days). In all experiments, the rats were anesthetized with Zoletil (tiletamine with zolazepam, 40 mg/kg, IP; 50 mg/kg, IP) and xylazine (10 mg/kg, IP) and for repair evaluation of the liver, blood and liver samples were collected for histopathological examination. During the periods of induction of liver fibrotic or necrotic injury and intraperitoneal treatment, the rats were carefully monitored for clinical signs of pain, salivation and abnormal behavior.

### 2.7. Histopathological Analysis

Two weeks after pure HAn, AHAna, or Asta treatment, the rat livers were harvested from each group and were fixed with 10% buffered neutral formalin solution. The specimens were then dehydrated in graded ethanol solutions, cleared in xylene and embedded in paraffin. Five mm thick sections were prepared and hematoxylin and eosin (H&E) dyes were used to carry out the staining for histopathological examination.

### 2.8. Alanine Aminotransferase and Aspartate Aminotransferase Assays

Alanine aminotransferase (ALT) relatively accurately reflects liver injury or disease because ALT is principally produced in the liver. Elevated ALT levels are usually correlated with liver cell damage, such as liver injury and hepatitis. Aspartate aminotransferase (AST) is present in the mitochondria of liver cells. Assessing other analytical indexes to accurately diagnose liver damage is necessary. ALT and AST were measured to assess the repair of the injured livers before and after AHAna or pure HAn injections. Serum samples were prepared in accordance with the instructional protocol (AAT Bioquest, Sunnyvale, CA, USA). Each 100-μL mixture of the sample and ALT or AST enzyme on the plate was incubated at 37 °C for 20–30 min in the dark. Absorbance was determined on a microplate reader at 575 nm (Thermo Scientific, Waltham, MA, USA) (*n* = 3).

### 2.9. Statistical Analysis

The data are expressed as mean ± standard error of the mean. The in vitro experimental data were pooled from three independent tests. The results were analyzed through one way analysis of variance using SPSS version 17.0 to identify the existence of significant differences (*p* ≤ 0.05) between experimental groups.

## 3. Results and Discussion

### 3.1. Observation of Asta-HA Nano-Aggregates (AHAna)

**Figure 2:**
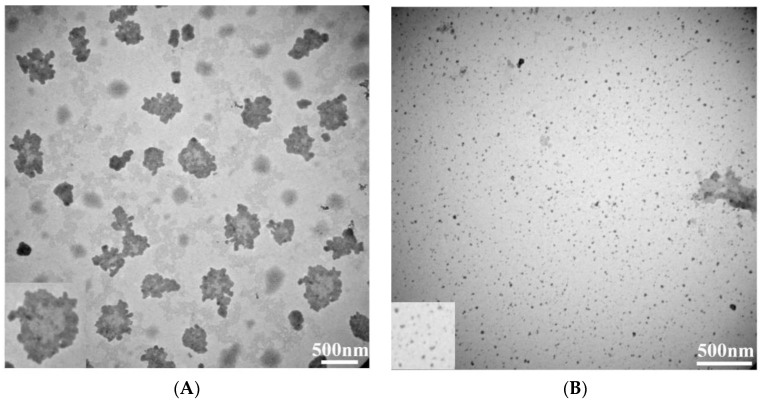
Transmission electron microscopy (TEM) images of the fabricated AHAna aggregates (**A**) and HAn nanoparticles (**B**).

The EFS has been used in our laboratory to prepare biopolymeric nanoparticles. The EFS provides an electrostatic force for controlling HA molecules as they undergo self-assembly to form a nanoparticle shape. Figure 1 presents the schematic procedures for preparing AHAna through the EFS by applying the designed preparation parameters. Transmission electron microscopy images showed that the prepared HAn were effectively dispersed in solution and had a spherical shape, with a mean diameter of approximately 15 ± 2.1 nm (Figure 2). When the hydrophobic Asta drug was incorporated into the HAn production process, the HA molecules were glued or aggregated to form a loose spherical structure (before crosslinking treatment) with Asta dissolved in DMSO (a bipolar solvent), which caused Asta molecules to adhere to the surface and be incorporated into the matrices of the HA loose nanoparticles. Consequently, the hydrophilic domain of Asta bound itself onto the hydrophilic sites or regions of the HA loose spheres. Simultaneously, the hydrophobic region of Asta was bound, in the form of a thin layer but in a continuous phase, to the hydrophobic regions of the HA nanoparticles. Thus, the Asta component brought together the individual HAn to form aggregates (AHAna) and produce clusters of irregular shapes and increasing sizes with gross diameters of approximately 210–500 nm (Figure 1).

### 3.2. Characteristics of AHAna

**Figure 3:**
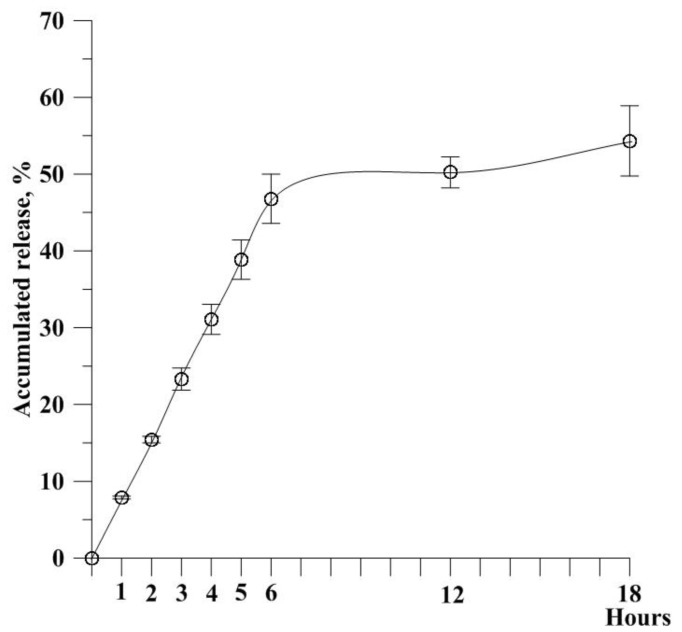
In vitro release profile of Astaxanthin (Asta) from AHAna aggregates in phosphate buffered saline (PBS) (pH 7.4). Values are mean ± standard error of the mean (SEM).

The IE of the fabricated AHAna was approximately 93%. As shown in TEM images, Asta was likely incorporated within the HAn in substantially aggregate mode and thus exhibited a relatively high IE (Figure 2). Figure 3 illustrates the release profile of Asta from AHAna, with rapid release (approximately 47%) within the first 6 h of incubation. A relatively slow release profile was exhibited during 6–18 h incubation and approximately 54% Asta was released from AHAna after 18 h-incubation. The low and slow release of AHAna can probably be attributed to the low solubility of Asta and precipitate when coming into contact with the aqueous solution.

### 3.3. Assessment of Cell Viability

**Figure 4:**
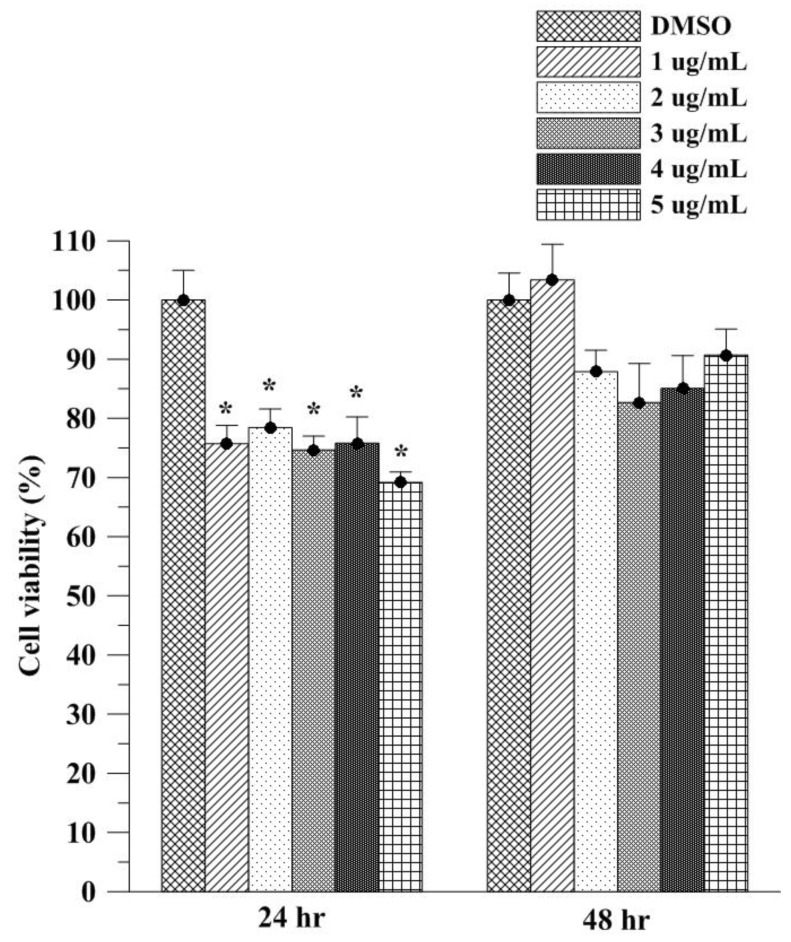
Cell viability analysis of different Asta amount dissolved in dimethyl sulfoxide (DMSO) by MTT assay in L929 normal fibroblasts. Slight and significant decreases in cell viability were found at 24 h as the reduction was minified or eliminated after 48 h incubation. Values are mean ± SEM (*n* = 3; * *p* < 0.05 by ANOVA).

The in vitro effects of Asta on cell viability were examined through MTT assay (Figure 4). After 24 h exposure, Asta at concentrations ranging from 1 to 5 μg/mL led to significant decreases of approximately 20–30% in L929 normal fibroblasts. The decrease of cell viability was weaker after 48 h of exposure to Asta (15–10%). Furthermore, 1 μg/mL Asta caused a slight and non-significant increase in cell viability in L929 cells. L929 cell viability at 48 h after 1 and 5 μg/mL Asta treatment in the current study was similar to the findings of primary retinal cells [17], indicating that Asta dissolved in DMSO slightly reduces or increases cell viability in normal cells. Slight cell viability decreasing the effects of Asta in DMSO after 24 and 48 h exposure rationalizes the aggregation use of Asta by HAn. In our previous study, HAn at concentrations between 10 and 40 μg/mL did not inhibit cell viability in L929 normal fibroblasts [12]. Encapsulation of natural compounds by using HAn has also been found to decrease the cell viability inhibitory effects of pure compounds in L9292 normal cells [13]; this further highlights the advantages of HAn aggregation and encapsulation. DMSO has been demonstrated to inhibit early growth response gene expression, arrest mouse embryo fibroblasts in the cell cycle and damage mitochondrial integrity and membrane potential in murine astrocytes [18,19]. By contrast, Asta has been found to not only protect against oxidative stress in mononuclear cells but also to exhibit free radical–scavenging, singlet oxygen quenching and antioxidant activities in various cell lines, such as PON1-Huh7, Huh7 and HepG2 cells [20,21]. Therefore, the shift L929 viability between 24 and 48 h incubation may be because Asta dissolved in DMSO leads to the weak inhibition of early growth response gene expression and slight arrest in the cell cycle at 24 h, followed by the free radical scavenging and oxidative stress-protective effects of Asta to restore the cell viability of L929 fibroblasts.

### 3.4. Histopathological Analysis of Rat Livers Treated with AHAna

**Figure 5:**
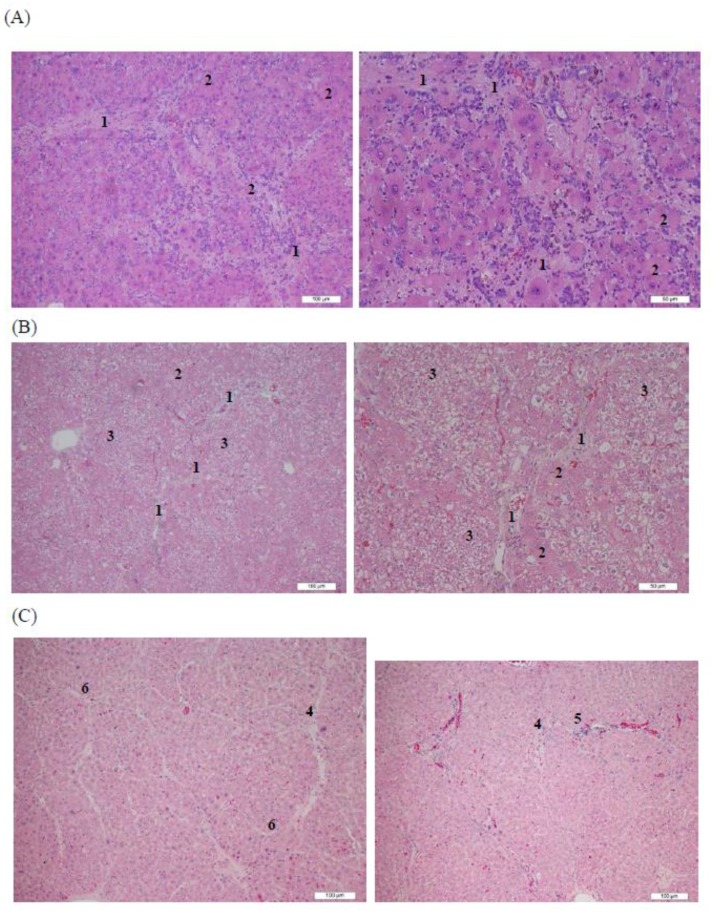

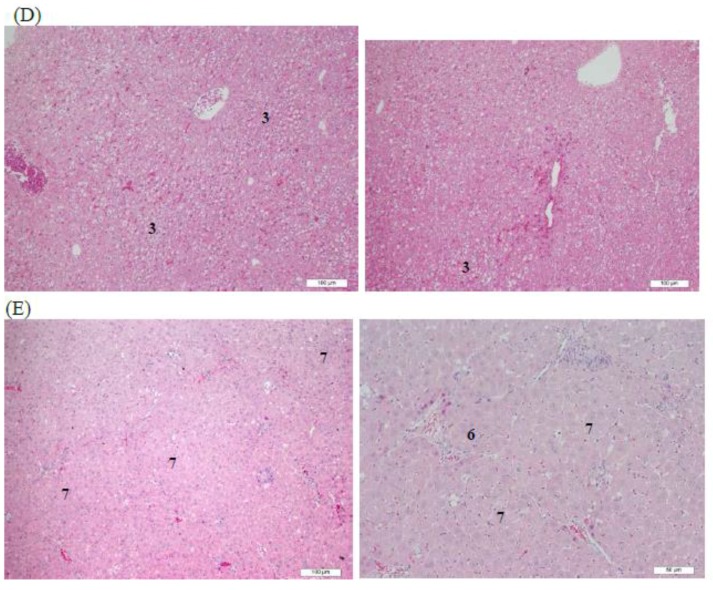
Histopathological analysis of retrorsine-CCl_4_-induced rat liver fibrosis and necrosis in the presence or absence of injective treatments. (**A**) Severe liver fibrosis (**1**) and necrosis/degeneration of hepatocytes (**2**) were observed before the treatments injected; (**B**) Obvious liver fibrosis (**1**) and necrosis/degeneration of hepatocytes (**2**) as well as numerous fatty degeneration-like areas (**3**) were seen without treatment after 2-week (negative control); (**C**) Liver fibrosis/necrosis was clearly decreased in most areas compared to negative control while in a few areas the fibrotic tissues (**4**), inflammatory cell infiltration (**5**) and some formation of pseudolobules (**6**) were seen after 2 week treatment with HAn injection; (**D**) Liver fibrosis and necrosis were not observed as some fatty degeneration-like areas (**3**) were found after AHAna injection for 2 weeks; (**E**) Liver fibrosis and necrosis were reduced whereas swelling of hepatocyte (**7**) and formation of pseudolobules (**6**) were observed after 2 week injection of pure Asta.

Rat liver tissues exposed to retrorsine and CCl_4_ before the treatments exhibited severe fibrosis and hepatocyte necrosis and degeneration (Figure 5A). In week 2, the rat liver tissue in the negative control group exhibited numerous fibrotic and fatty degeneration-like areas (Figure 5B). In the group treated with HAn injection, a decrease in hepatocyte swelling was observed but clear fibrotic tissues remained around the central vein and hepatic lobules of the rat liver (Group B, Figure 5C). By contrast, fibrotic tissues were not observed in the rat livers that received an AHAna injection, although some fatty degeneration-like areas were observed (Group C, Figure 5D). In the group injected with Asta, obvious swelling of the hepatocytes, remaining fibrotic tissues and fibrocytes was observed. Additionally, the hepatic lobules were divided into several pseudolobules by the remaining fibrotic tissues at high magnification (Group D, Figure 5E). Overall, the intraperitoneal injections of both AHAna and HAn for 2 weeks (Group B and C) resulted in an obvious improvement in liver fibrosis. The hepatic tissues treated with 2 weeks of injection of Asta had somewhat diminished fibrotic tissues and exhibited formation of pseudolobules and swelling hepatocytes (Group D).

### 3.5. ALT an AST Analysis of Rat Sera

**Figure 6:**
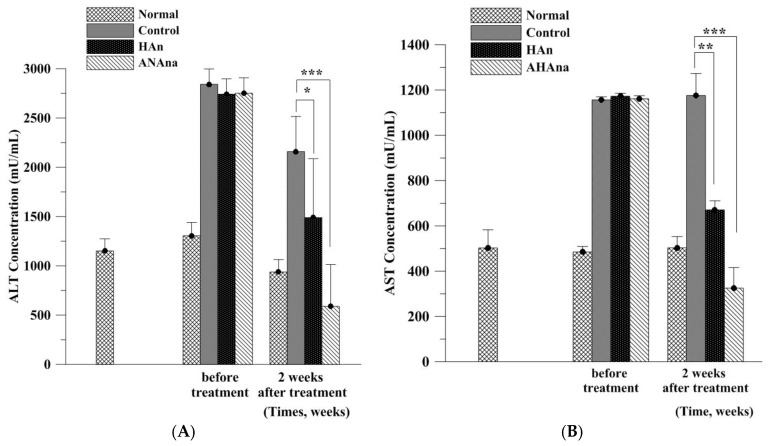
Serum biochemical analysis of liver function for the rats in the presence or absence of HAn and AHAna treatment. Comparison of serum alanine aminotransferase (ALT) (**A**) and aspartate aminotransferase (AST) (**B**) level between experimental groups and negative control was assessed. Both injective HAn and AHAna treatments reduced ALT and AST values while AHAna injection exhibited the strongest effects on hepatic function improvement in terms of serum biochemical measurement. Values are mean ± SEM (*n* = 3; * *p* < 0.05, ** *p* < 0.01 and *** *p* < 0.001, by ANOVA).

AST and ALT levels were 485.0 and 1304.81 mU/mL, respectively, before liver fibrosis and necrosis were induced in the rats (Figure 6; Group A). AST and ALT levels were significantly elevated in the negative control group in week 2 (Group B) compared with the levels before the injury was induced in the livers. AST and ALT levels reached 1156.36–1176.72 and 2741.7–2841.7 mU/mL, respectively, in Group B, indicating that the liver tissues incurred severe injury and damage. In week 2, the administration of HAn injections resulted in significant decreases in both ALT and AST levels (670.74 and 1489.91 mU/mL, respectively) in Group C compared with those in Group B, implying that hepatic repair occurred in the rat livers to some extent. By contrast, the group that received AHAna injections (Group D) had the largest reduction in both ALT and AST levels (325.11 and 588.4 mU/mL, respectively) compared with the HAn injection and no-treatment groups, indicating that AHAna had the strongest restorative effect on liver fibrosis and necrosis. Liver-protective effects of Asta have been reported in recent years. Studies have shown that Asta can reverse diet-induced insulin resistance and steatohepatitis in mice because Asta has been implicated as a potential therapy for NASH [22]. 

The imbalance between the cellular pro-oxidant and antioxidant redox favoring the pro-oxidant state can result in oxidative stress and cause potential cellular damage [23]. The sources of oxidative stress include elevated reactive oxygen species (ROS), reactive nitrogen species or reduced production of antioxidants. ROS is crucial in the occurrence of liver damage and initiation of hepatic fibrogenesis, while it can stimulate profibrogenic mediator production from Kupffer cells and circulating inflammatory cells [24]. ROS can further directly activate hepatic stellate cells, leading to the onset of liver fibrosis. Liposomal Asta (10 mg/kg BW per day) has been found to nearly completely rescue the largely dropped hepatic SOD, catalase and GSH-Px induced by lipopolysaccharide (LPS) in vivo, strongly suggesting that encapsulation of liposomes can enhance the antioxidative effects of Asta against oxidative stress in the liver. The study by Chiu et al. shed some light on the potential therapeutic and preventive effects of Asta and nanoparticles-encapsulated Asta against liver fibrosis [5]. Yang et al. indicated that Asta prevents TGFβ1-induced profibrogenic gene expression by inhibiting Smad3 activation in hepatic stellate cells, suggesting that Asta is a preventive and therapeutic alternative that prevents hepatic fibrosis [25]. The preventive and reversal effects of Asta on primary hepatic stellate cells have also been demonstrated [26]. Our histopathological and serum biochemical analysis results revealed that AHAna injection had the most substantial antifibrotic and hepatic injury–reparative effects among the experimental groups, which corresponds to the aforementioned previous findings. However, HAn injection also had somewhat restorative effects on the fibrotic and necrotic liver in the rodent retrorsine–CCl_4_ model. This is unsurprising because HA has been revealed to possess antifibrotic characteristics. Liver fibrosis or cirrhosis has been reported to be correlated with several crucial factors, such as the increased generation of oxygen free radicals and lipid peroxidation and the activation of hepatic stellate cells [7,27]. HA or hyaluronic acid has been found to inhibit hepatic stellate cell adhesion in vitro despite an increase in DNA synthesis [28]. Moreover, HA and its fragments have been shown to reduce the amount of lipid peroxidation secondary products on liposomal skin lipid models [29]. Additionally, low molecular-weight hyaluronic acid can prevent oxygen free radical damage to granulation tissue during wound healing and is therefore effective as a native compound against oxygen free radicals [30]. These antifibrotic characteristics correspond to our histopathological results for Group C, in which liver fibrosis residues can be observed but fibrosis and hepatocyte swelling severity were clearly decreased. The decline in the ALT and AST levels of the HAn injection group is understandable because HA has been revealed to exert antifibrotic and lipid peroxidation–reducing actions in the present and previous studies. Additionally, this supports that HA should be selected as the biomaterial for fabricating the nanoparticles for Asta aggregation in the current study, in particular for liver repair and fibrosis elimination. In addition to HA aggregation, liposome-encapsulation has also been utilized to efficiently improve the inflammation-alleviating and antioxidative effects of Asta against LPS-induced elevated oxidative stress and acute hepatotoxicity. Therefore, it seems that HA nanoparticles and liposomes are the two alternative biomaterial approaches for Asta delivery in hepatic therapeutics [5].

Additionally, the observation of the remaining fibrotic tissues and swelling hepatocytes in Group C (HAn injection) indicated that the reparative capacity of the HAn was not alone sufficient for the chemical toxicant-induced fibrosis and necrosis in the liver; therefore, the combined restorative effects provided by AHAna had the most substantial effect on liver fibrosis and necrosis in Group D. When the retrorsine–CCl_4_-induced hepatic fibrotic and necrotic tissues were treated with Asta injection alone (Group E), the fibrotic tissues decreased compared with those without treatment. However, pseudolobule formation and some hepatocyte swelling were observed. This may be because the Asta compounds are insufficiently water soluble and dispersible in aqueous solution and must be dissolved in the organic solvent DMSO. Intraperitoneal injection of DMSO-dissolved Asta simultaneously had fibrosis-inducing effects (the solvent DMSO) and fibrosis-easing activities (Asta), resulting in the formation of some pseudolobules but a decrease in fibrotic tissues in the rat liver. AHAna injection, despite some fatty degeneration-like areas, the remaining fibrotic tissues, pseudolobules or hepatocyte swelling were not found, indicating that AHAna alone can overcome the drawbacks of using HAn or Asta alone through successfully restoring prior ALT and AST levels and remedying liver fibrosis.

## 4. Conclusions

Asta was successfully aggregated with HA nanoparticles and the EFS to conduct AHAna preparation in the current study. The fabricated AHAna, with high EE and slow-release profiles, had the strongest reparative effect on retrorsine–CCl_4_-induced liver fibrosis and necrosis in the rat model compared with the HAn and Asta treatment and the negative control. The findings suggest that AHAna is a potential therapy for toxicant-caused liver injury.
